# Unraveling epigenetic heterogeneity across gastrointestinal adenocarcinomas through a standardized analytical framework

**DOI:** 10.1002/1878-0261.13772

**Published:** 2024-12-18

**Authors:** Rita Pinto, Hege Marie Vedeld, Guro Elisabeth Lind, Marine Jeanmougin

**Affiliations:** ^1^ Department of Molecular Oncology, Institute for Cancer Research Oslo University Hospital – Norwegian Radium Hospital Oslo Norway; ^2^ Department of Biosciences, The Faculty of Mathematics and Natural Sciences University of Oslo Norway

**Keywords:** differential variability, DNA methylation, molecular subtyping

## Abstract

In this study, we propose an alternative approach for stratifying genome‐scale DNA methylation profiles of gastrointestinal (GI) adenocarcinomas based on a robust analytical framework. A set of 978 GI adenocarcinomas and 120 adjacent normal tissues from public repositories was quality controlled and analyzed. Hierarchical consensus clustering of the tumors, based on differential epigenetic variability between malignant and normal samples, identified six distinct subtypes defined either by a pan‐GI or a lower GI‐specific phenotype. In addition to methylation levels, aberrant methylation frequencies and the degree of DNA methylation instability contributed to the characterization of each subtype. We found significant differences in the outcome of patients, with the poorest overall survival seen for those belonging to a pan‐GI subtype with infrequent aberrant methylation. In conclusion, our standardized approach contributes to a refined characterization of the epigenetic heterogeneity in GI adenocarcinomas, offering insights into subtype‐specific methylation with the potential to support prognostication.

## Graphical abstract

This study shows that patients with gastrointestinal adenocarcinomas can be stratified into six distinct subtypes based on genome‐scale DNA methylation profiling. This stratification, achieved using a robust analytical framework, aligns with established tumor characteristics and holds potential to support prognostication.

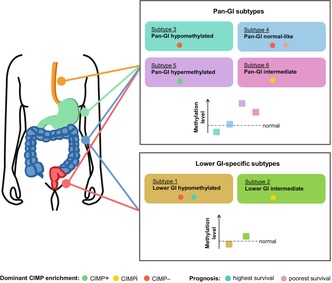

**Abbreviations**
CI: confidence interval
CIMP: CpG island methylator phenotype
COAD: colon adenocarcinoma
CRC: colorectal cancer
DMI: DNA methylation instability
DVP: differentially variable probe
EBV: Epstein–Barr virus
ESCA: esophageal carcinoma
FDR: false discovery rate
GEO: Gene Expression Omnibus
GI: gastrointestinal
HR: hazard ratio
HM450: Illumina Infinium HumanMethylation450
NMDS: non‐metric multidimensional scaling
READ: rectum adenocarcinoma
STAD: stomach adenocarcinoma
TCGA: The Cancer Genome Atlas

## 1 Introduction

The World Health Organization classification of adenocarcinomas arising in the gastrointestinal (GI) tract mostly relies on tissue or organ of origin and histologic type [1]. Alongside the TNM staging system, these classifications remain the benchmark for prognostication and treatment decisions. However, recent technological developments have allowed the introduction of clinically relevant molecular phenotypes [2, 3, 4, 5, 6], adding new dimensions to the traditional classification systems. While most of the existing molecular stratifications are organ‐specific, integrative analyses of multi‐platform data across tumor types identified GI adenocarcinomas as one distinct group with common molecular features spanning anatomic boundaries [7, 8]. Such features mirror a shared endodermal origin and exposure to similar environmental factors. A comprehensive molecular characterization of GI adenocarcinomas as a whole has recently enabled refining tumor classification into five GI mixed molecular subtypes characterized by (1) chromosomal instability, (2) genomic stability, (3) microsatellite instability, (4) Epstein–Barr virus (EBV)‐positive, and (5) hypermutated‐single‐nucleotide variant predominant [8]. These subtypes reflect broad biological processes which are not confined to a single tumor location. However, although some of these groups are well‐established in individual tumor types [9, 10], their clinical value in GI adenocarcinomas as a whole has not yet been demonstrated.

Examination of DNA methylation in individual GI adenocarcinoma types has also revealed relevant epigenetic patterns, including the CpG island methylator phenotype (CIMP) [3, 4, 11, 12, 13, 14, 15], well‐established in colorectal cancer (CRC), and characterized by frequent hypermethylation at CpG islands. Despite the potential clinical relevance of DNA methylation classification of GI adenocarcinomas, only a few array‐based DNA methylation profiling studies include associations with clinical and molecular data [8, 16]. In their work, Sánchez‐Vega et al. focused on extending CIMP stratification across GI adenocarcinomas [16] and showed that tumors tend to cluster by DNA methylation level similarities rather than by organ of origin. Another study from Liu et al. revealed distinct GI subtypes sharing similar epigenetic patterns independently of CIMP status [8], suggesting that there is room for an improved DNA methylation stratification. However, consistent and robust workflows to identify such subtypes are lacking, challenging the description of epigenetic heterogeneity across GI adenocarcinomas and the potential for clinical implementation.

Taking advantage of large‐scale data available from The Cancer Genome Atlas (TCGA) project and Gene Expression Omnibus (GEO), and applying a robust analysis framework, we here investigated the epigenetic heterogeneity across GI adenocarcinomas. We aimed to present the common and distinct DNA methylation profiles and to identify meaningful clinical features associated with GI subtypes, by looking beyond organ of origin similarities or hypermethylation at CpG islands only.

## 2 Materials and methods

An overview of data preparation and analyses is shown in Fig. S1.

### 2.1 Sample selection and DNA methylation data acquisition

DNA methylation data generated from Illumina Infinium HumanMethylation450 (HM450) BeadChip were obtained from NIH Genomic Data Commons Legacy Archive in November 2019 as part of TCGA project (dbGaP accession number: phs000178.v11.p8). TCGAbiolinks package [17, 18] (v2.14.0) was used for query and download of esophageal carcinoma (ESCA), stomach adenocarcinoma (STAD), colon adenocarcinoma (COAD), and rectum adenocarcinoma (READ) TCGA datasets, both from ‘primary solid tumor’ and ‘solid tissue normal’ sample categories. Data were downloaded in the form of IDAT files containing the raw intensity signals. ESCA samples that were not esophageal adenocarcinomas or histologically normal adenocarcinoma‐adjacent tissue were not considered. Due to mismatches in sample IDs, 25 COAD samples were also removed from the dataset.

Sample processing has been described in the original TCGA papers [2, 3, 4]. Clinical annotations obtained from the different study groups were also downloaded. The set of available annotations varied across cancer types and across samples within each cancer type.

Additional raw DNA methylation data from 125 esophageal adenocarcinomas, 43 esophageal squamous mucosa, and 21 gastric normal samples were obtained from GEO (accession number GSE72872) [12].

Probe annotation, including relation to annotated genes and to CpG islands, was obtained using the IlluminaHumanMethylation450kanno.ilmn12.hg19 package [19] (v0.6.0).

All the data were read into R using the minfi package [20] (v1.32.0).

### 2.2 Statistical analyses and graphical representations

Quality control, preprocessing, and statistical analyses were conducted with R software versions >3.6, and performed on *M*‐values as previously recommended [21], unless otherwise stated. Correlation between continuous variables was analyzed by Spearman's rank correlation test, whereas association between categorical variables was analyzed by Fisher's exact test. For multiple group comparisons, Tukey's Honest Significant Difference test was performed together with ANOVA. A *P*‐value ≤0.05 was considered significant.

For representation of the results, the R packages ggplot2 [22] (v3.3.5), venndiagram [23] (v1.7.1), enhancedvolcano [24] (v1.12.0), and complexheatmap [25] (v2.10.0) were used. Non‐metric multidimensional scaling (NMDS) plots were drawn using the function *metaMDS* implemented in vegan package [26] (v2.5.7).

### 2.3 Quality control and preprocessing

#### 2.3.1 Quality control

Quality control of the dataset was performed using the minfi package (v1.32.0). For each probe, a detection *P*‐value comparing the signal intensity difference between the analytical probes and a set of negative control probes on the array was calculated. Poor quality samples, with mean detection *P*‐value >0.01, were discarded. Additional probe quality control measures available in minfi were performed (data not shown).

#### 2.3.2 Normalization

Datasets from each tumor location (including TCGA and GEO datasets) were normalized separately by functional normalization [27] (Fig. S2). Normalized data from all tumor locations were then combined in one single pan‐GI dataset.

#### 2.3.3 Filtering of probes

A total of 485 577 CpGs are queried by the HM450 BeadChip array [28]. Filtered‐out probes included, in sequential order, (1) 65 explicitly built‐in probes meant to interrogate SNPs and not methylation status (as an internal control) [29] are excluded by minfi during quality control by default, (2) 101338 probes with detection *P*‐value >0.01 in at least one sample, (3) 8160 probes mapped to the X or Y chromosomes, (4) 10450 probes containing a common SNP with annotated minor allele frequency ≥1% in the CpG site itself or in the single‐base extension site, within 10 bp of the target cytosine [29], (5) 20708 probes aligned to multiple regions of the genome [30], and (6) 194 probes with at least one infinite *M*‐value, which is not accepted in batch correction. This filtering resulted in 344 662 features, that is, ~71% of the initial probes, comparable with other studies [11, 31].

#### 2.3.4 Batch correction

A batch was defined by the column ‘Slide’ available from TCGA sample annotation. Multiple batches were documented within each cancer type. Singular value decomposition analysis—implemented in champ package [32, 33] (v2.20.1)—revealed that, when tumor site and sample type (tumor/normal) were accounted for, a significant batch effect remained. In order to adjust for technical heterogeneity, batch correction was performed using ComBat, an empirical Bayes‐based method implemented in the sva package [34] (v3.42.0).

### 2.4 Final dataset

After quality control and preprocessing, further sample exclusion consisted of one ESCA normal sample clustering with its tumor counterpart, as well as two STAD normal samples impairing patient effect estimation. A final dataset consisting of 1098 samples (978 tumors and 120 normal samples) was considered for downstream analyses (Table S1). Demographic data for patients are as follows: esophageal adenocarcinoma (88% male, median age 65 years, range 27–87 years); gastric adenocarcinoma (66% male, median age 67 years, range 30–90 years); colonic adenocarcinoma (51% male, median age 68 years, range 31–90 years); and rectal adenocarcinoma (54% male; median age 64 years, range 31–90 years).

### 2.5 Identification of differentially variable CpG probes

Differential variability of methylation levels between tumor and normal samples was tested using the ‘Diffvar’ method [35], available in missmethyl package [36] (v1.28.0) and based on the statistical framework of limma [37]. The analyses were carried out independently for each tumor location, accounting for patient effect. *P*‐values were adjusted for multiple testing using the false discovery rate (FDR) criterion and Benjamini–Hochberg procedure. A probe was defined as differentially variable (DVP) if it met the criteria of a FDR corrected *P*‐value <0.05 (Fig. S3A). If the methylation variability of a DVP was higher in tumor than in normal samples we defined it as hypervariable, otherwise it was considered as a hypovariable probe. No restrictions on variability ratio were applied.

An overview of the number of significant DVPs found (either tumor‐specific or shared across the different tumor types) is represented in Fig. S3B and in Table S1.

### 2.6 Unsupervised hierarchical clustering analysis based on DNA methylation

A set of 20 663 probes representing the union of the 5% most significant DVPs found in each cancer type was used as input for the unsupervised hierarchical clustering. The optimal number of clusters was assessed based on resampling 80% of the probes and tumor samples over 2000 iterations of hierarchical clustering for *k* = 2 to *k* = 10 using the Euclidean distance as a metric for clustering and Ward's method for linkage as implemented in the consensusclusterplus package [38] (v1.50.0). The optimal number of clusters was determined based on the visual inspection of the output plots (Fig. S4) by using the following criteria: (1) the area under the cumulative distribution function curve did not increase significantly (Fig. S4A,B) and (2) the stability of sample clustering was high (Fig. S4C).

### 2.7 Coefficient of epigenetic heterogeneity

The degree of epigenetic inter‐tumor heterogeneity was defined as the distance between dichotomized β‐values of any two samples, based on the set of DVPs used as input for the hierarchical clustering. The β‐value threshold for dichotomizing the data was 0.2 (β‐value ranging from 0 to 1, being ‘0’ completely unmethylated and ‘1’ completely methylated).

### 2.8 Determination of CIMP status

The CIMP status of each sample was determined based on a list of 89 signature loci as suggested previously [39]. Out of these 89 probes, 14 were filtered out during preprocessing, leaving 75 probes for analysis. For each individual tumor type, tumor samples were labeled as CIMP+, CIMP–, or CIMPi based on the β‐values mean across the set of selected probes. Labels were assigned using k‐means clustering with *k* = 3 classes on the vector of the mean β‐values with length equal to the number of tumor samples within each tumor type. The centroids for each of these three classes were defined as the first, second, and third quartiles of the distribution of the mean methylation values.

### 2.9 *MLH1* promoter hypermethylation calling

*MLH1* promoter hypermethylation was determined as previously described [40]. Briefly, normalized β‐values from four sites associated with loss of *MLH1* expression were analyzed. If all the four probes were hypermethylated according to the definition of Benhamida et al. [40], the sample was labeled as having *MLH1* promoter hypermethylation.

### 2.10 Estimation of DNA methylation event frequencies and instability

The frequencies of hyper‐ and hypomethylated probes were calculated based on the RESET algorithm [41]. Probes with low or high β‐values (mean <0.1 or >0.8, respectively) and standard deviation lower that 0.005 in normal samples were selected. Based on this set of probes, RESET algorithm defined hyper‐ and hypomethylated probes in tumor samples. Frequencies were then estimated as the percentage of probes found aberrantly methylated compared to normal tissues.

The degree of aberrant methylation in each sample was determined by a DNA methylation instability (DMI) score in accordance with Saghafinia et al. [41]. DMI scores combine the frequency of both hyper‐ and hypomethylated loci using the F_β_‐measure. We used β = 1 for balanced distributions of hyper‐ and hypomethylation frequencies.

### 2.11 Survival analysis

The survival analyses were performed using the Survival [42, 43] (v3.2.13) and bootstepaic [44] (v1.2.0) packages. Five‐year overall survival, estimated from the annotation file for the GEO cohort or calculated from time of surgery until death of any cause for the TCGA cohort, was used as endpoint. Patients who did not have an event occurrence (death) were censored at last follow‐up. Data from 957 out of 978 patients were available for analysis.

Kaplan–Meier analysis with the log‐rank test was performed to evaluate overall survival differences among groups. The effect of a set of variables (GI subtype, tumor localization, gender, age, stage, CIMP status, *MLH1* promoter methylation status, and DMI score) on survival was independently evaluated by univariate Cox's proportional hazard model. Hazard ratios (HRs) and 95% confidence intervals (CIs) were derived from the model, and significance of the parameters was assessed using Wald's test.

A multivariate approach was also run based on Cox's proportional hazard model and preceded by a stepwise selection procedure by Akaike information criterion, in order to identify a subset of relevant predictor variables from the set of available clinicopathological data. To ensure robustness in the selection procedure, a bootstrap approach with 1000 iterations was implemented. Due to known associations with patient prognosis, CIMP status was also included in the final model even though not selected by this procedure. A total of 790 patients had information on all predictor variables and were included in the multivariate analyses. Again, HRs and 95% CIs were derived from the model, and significance of the variables included in the final model was assessed using Wald's test. To evaluate the assumption of proportionality, a chi‐square test was performed. For all statistical analyses, differences were considered statistically significant at *P*‐value < 0.05.

## 3 Results

### 3.1 Epigenetic heterogeneity is linked to GI tract location and global methylation differences

**Fig. 1:**
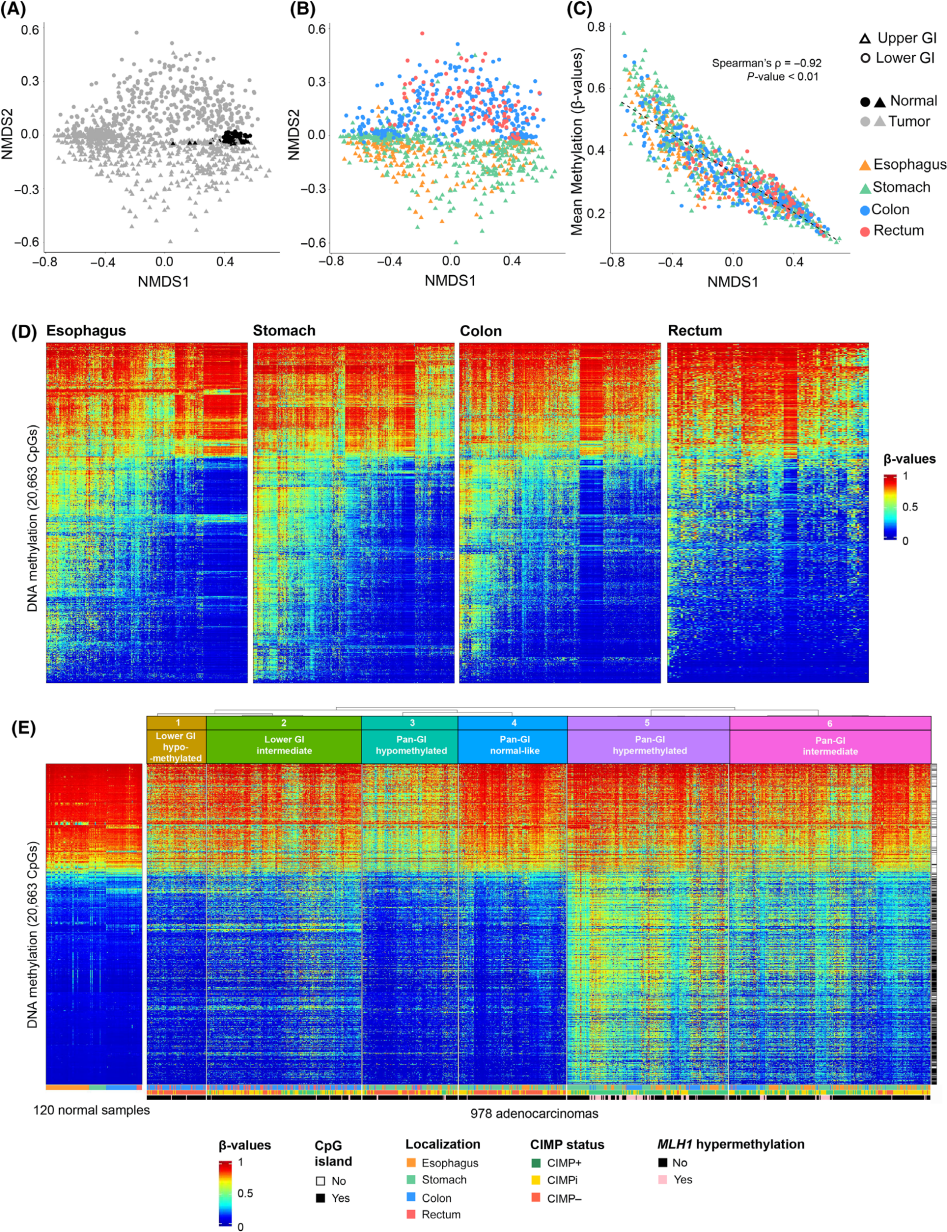
Epigenetic heterogeneity of gastrointestinal (GI) adenocarcinomas from TCGA and GEO repositories. (A) and (B) non‐metric multidimensional scaling (NMDS) plots based on the union of the 1% most significant differentially variable probes (DVPs) found for each tumor type. Each dot represents one sample. (C) Correlation between the first axis of NMDS and the mean methylation for each tumor sample. The Spearman correlation coefficient (ρ) and associated *P*‐value are indicated. (D) Clustering of samples in individual tumor types. The samples (in columns) and a set of 20 663 probes (in rows) representing the union of the 5% most significant DVPs (hyper‐ or hypo‐) found for each tumor type were clustered based on β‐values using the Euclidean distance metric and Ward's linkage method. (E) Unsupervised hierarchical clustering of GI adenocarcinomas based on DNA methylation profiles. Samples are shown in columns. The optimal number of clusters was assessed based on 80% probe and tumor resampling over 2000 iterations using the Euclidean distance metric for clustering and Ward's method for linkage on *M*‐values. *M*‐values were converted to β‐values for representation purposes. The set of 20 663 probes (in rows) are displayed based on the order of unsupervised hierarchal clustering of the β‐values using the Euclidean distance metric and Ward's linkage method. An annotation bar to the right of the heatmap shows the content of probes localized within or outside CpG islands and bottom annotations show tumor localization, and CIMP and *MLH1* promoter methylation status. Normal samples clustered by localization are shown in the left panel. CIMPi, CIMP intermediate.

To explore the extent of epigenetic heterogeneity in GI tumors and normal samples, dimensionality reduction was carried out based on the union of the 1% most significant DVPs found for each tumor type. Most normal samples clustered together, in contrast to tumor samples, which displayed larger variability (Fig. 1A) as previously observed [45, 46]. Samples from the upper (esophagus and stomach) and lower (colon and rectum) GI tract were separated along the second axis of the NMDS (Fig. 1B), whereas the first axis correlated with mean methylation of the samples (Spearman's ρ = −0.92; *P*‐value <0.01) (Fig. 1C).

### 3.2 Consensus DNA methylation‐based clustering identifies pan‐GI subtypes

**Fig. 2:**
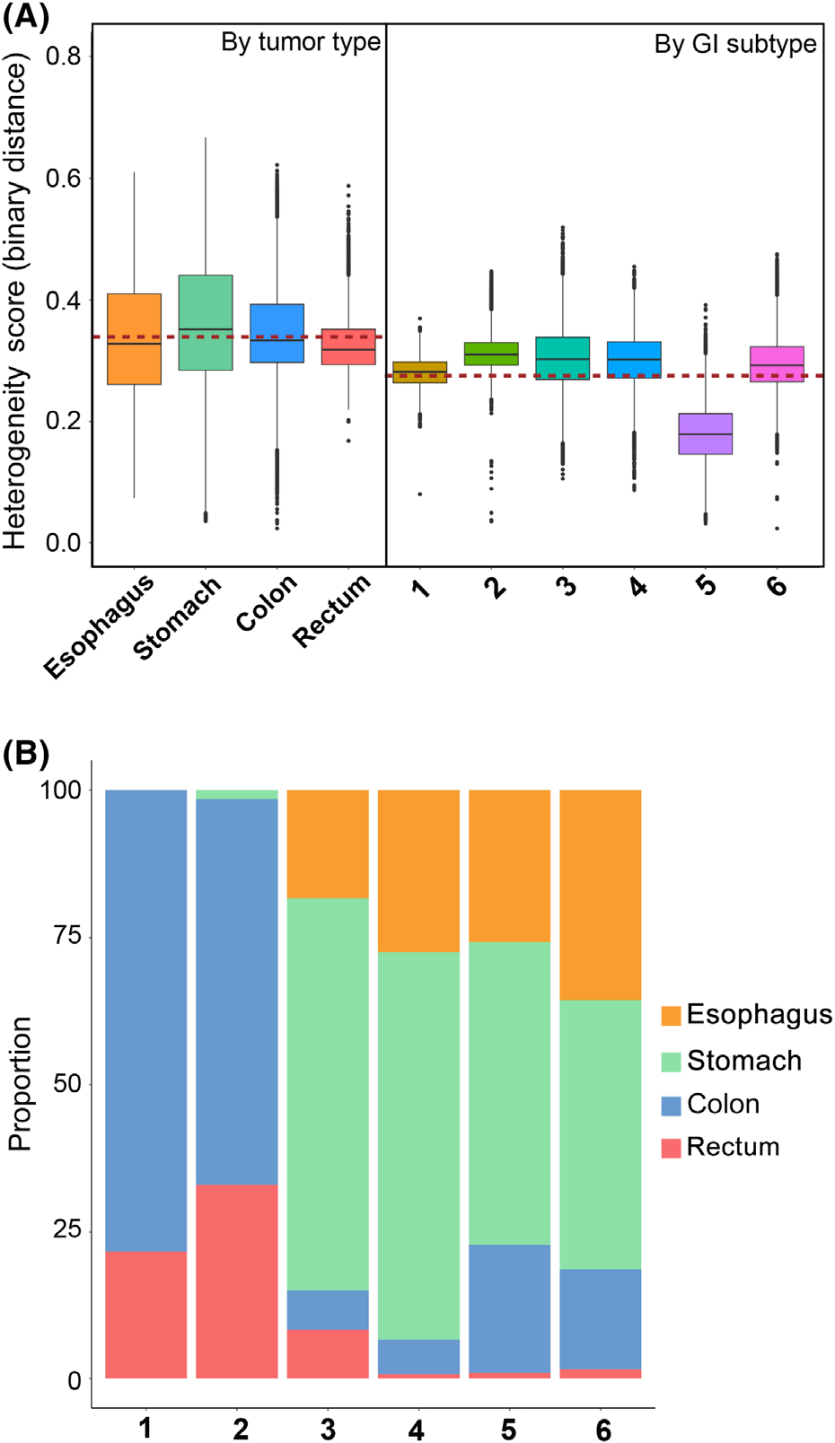
Epigenetic heterogeneity and distribution of organ of origin across the six DNA methylation‐based gastrointestinal (GI) subtypes. (A) Degree of epigenetic inter‐tumor heterogeneity quantified by computing binary distances between vectors of dichotomized β‐values of any two samples. The ends of the boxes and the middle line represent the lower and upper quartiles, and medians, respectively. The lines extending from both ends of the boxes indicate variability outside the lower and upper quartiles, with minimum/maximum whisker values calculated as Q1/Q3 −/+ 1.5 * interquartile rage (IQR). The average of the medians by tumor type (μ = 0.351) or by GI subtype (μ = 0.273) are labeled by a slashed red line. (B) Proportion of tumors from different anatomical locations in the GI tract across the six subtypes.

The heterogeneity observed across GI adenocarcinomas was further investigated within the individual tumor types (*i.e*., esophagus, stomach, colon, and rectum) (Fig. 1D). Similarities in DNA methylation patterns were found independently of the organ of origin. A consensus unsupervised hierarchical cluster analysis ran on the DNA methylation profiles of the whole set of tumors identified six stable and homogeneous subtypes (Fig. 1E; Fig. S4). The epigenetic heterogeneity was compared by calculating the binary distance between any two samples within each tumor type or subtype (Fig. 2A). The variability of the heterogeneity scores decreased, along with a relative decline in the average of the median values across the six subtypes (μ = 0.273) compared to the median values across the tumor types (μ = 0.351). These results confirmed an increase in the homogeneity of the subtypes compared to the individual tumor types (*P‐*value <0.01, by one‐way ANOVA test).

Subtypes 3 to 6 included tumors from all anatomical locations in the GI tract, suggesting that clustering of DNA methylation profiles goes beyond organ of origin (Fig. 2B). These four subtypes were therefore considered as ‘pan‐GI’ subtypes. Meanwhile, subtypes 1 and 2 were highly enriched in tumors of the lower GI tract (colon and rectum) and thus considered ‘lower GI‐specific’.

### 3.3 The GI subtypes exhibit distinctive DNA methylation profiles

**Fig. 3:**
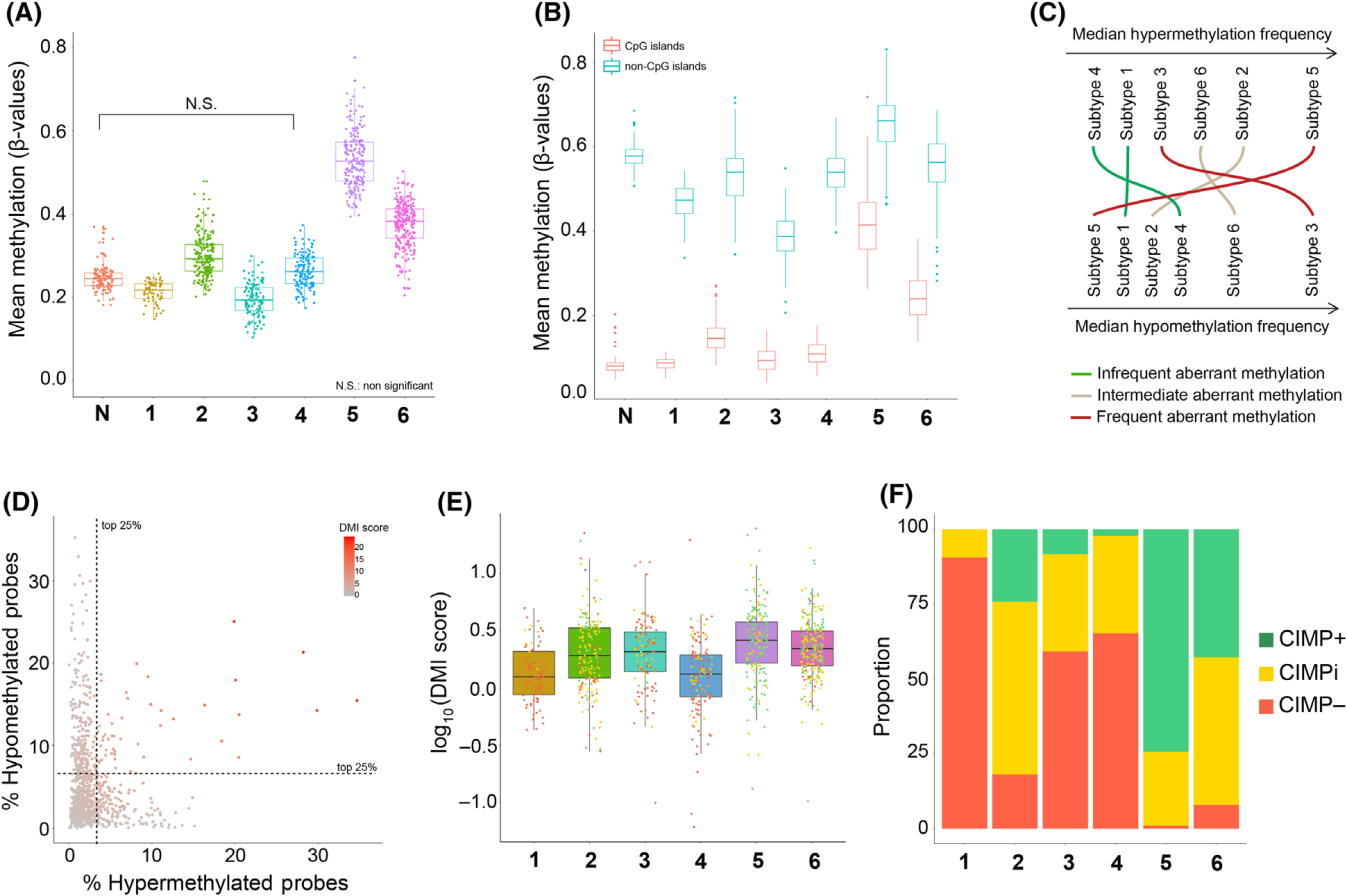
DNA methylation profiles and epigenetic instability across the six gastrointestinal (GI) subtypes. (A, B) distribution of mean methylation across samples in each subtype considering all the differentially variable probes (DVPs) used for clustering (A) or DVPs within or outside CpG islands independently (B). M‐values were converted to β‐values. In boxplots, the ends of the boxes and the middle line represent the lower and upper quartiles, and medians, respectively. The lines extending from both ends of the box indicate variability outside the lower and upper quartiles, with minimum/maximum whisker values calculated as Q1/Q3 −/+ 1.5 * interquartile rage (IQR). In (A), all the comparisons subtype *vs* normal (N) samples were significantly different (*P*‐value ≤0.05, by Tukey's Honest Significant Difference test), except for one (subtype 4 vs N; N.S.). (C) Methylation aberrancy across DNA methylation‐based subtypes. Comparison of the rank positions obtained for each subtype with respect to hypermethylation (top) or hypomethylation (bottom) event median frequencies according to Fig. S6. (D) Percentage of probes in each tumor sample that are hyper‐ (x‐axis) or hypomethylated (y‐axis) according to Fig. S6. Samples are color‐coded by DNA methylation instability (DMI) score. (E) DMI scores by subtype. Samples are color‐coded by CpG island methylator phenotype (CIMP) status according to (F). The ends of the boxes and the middle line represent the lower and upper quartiles, and medians, respectively, and the lines extending from both ends of the box indicate variability outside the lower and upper quartiles, with minimum/maximum whisker values calculated as Q1/Q3 −/+ 1.5 * IQR. (F) Distribution of sample CIMP status across the subtypes. CIMPi, CIMP intermediate.

The six GI subtypes were characterized by continuous variation in their DNA methylation levels as observed along the first axis of NMDS (Fig. S5). Subtype 5 displayed the highest mean DNA methylation levels, followed by subtype 6. These subtypes are hereafter referred to as ‘pan‐GI hypermethylated’ and ‘pan‐GI intermediate’, respectively. Subtype 3 showed significantly lower DNA methylation levels, whereas subtype 4 did not present significantly different DNA methylation levels compared to the set of normal samples (Fig. 3A). Accordingly, we have named these subtypes as ‘pan‐GI hypomethylated’ and ‘pan‐GI normal‐like’, respectively. Subtypes 1 and 2 represented the lower GI counterparts of the pan‐GI hypomethylated and pan‐GI intermediate subtypes, respectively, and therefore were named ‘lower GI hypomethylated’ and ‘lower GI intermediate’.

The differences in DNA methylation levels between the subtypes were particularly pronounced when examining probes within (*n* = 10 839) and outside CpG islands (*n* = 9824) (Fig. 3B). As expected [46], tumors exhibited increased methylation in probes mapped to CpG islands compared to normal samples, whereas a decrease in methylation levels was observed outside CpG islands. An exception was seen for the pan‐GI hypermethylated subtype 5, which showed increased DNA methylation levels independently of the genomic location of the probes.

### 3.4 Aberrant methylation frequencies and epigenetic instability further characterize the GI subtypes

The frequency of hyper‐ and hypomethylation events, indicating aberrant DNA methylation, varied among the six GI subtypes on a sample‐wise basis (Fig. S6). The pan‐GI hypermethylated subtype 5 and pan‐GI hypomethylated subtype 3 showed the highest frequencies of hyper‐ and hypomethylated events, respectively, (Fig. 3C) in accordance with the relative mean methylation values observed (Fig. 3A). Simultaneously, the pan‐GI hypermethylated subtype 5 exhibited low frequency of hypomethylated probes, while the pan‐GI hypomethylated subtype 3 showed low frequency of hypermethylated probes (Fig. 3C; Fig. S6).

DMI scores were calculated for each sample as a measure of the degree of aberrant methylation based on the frequencies of hyper‐ and/or hypomethylated probes (Fig. 3D,E). The lowest medians of DMI scores were found in the lower GI hypomethylated subtype 1 and in the pan‐GI normal‐like subtype 4. This is in agreement with their consistent infrequent aberrant methylation (Fig. 3C; Fig. S6) and the observation that the methylation levels in these subtypes closely resemble the methylation levels in normal samples (Fig. 3A). DMI was higher in the other four subtypes with no statistical differences between them. These observations suggested that high levels of either hyper‐ or hypomethylation (frequent aberrant methylation) contribute as much to epigenetic instability as the presence of both hyper‐ and hypomethylation simultaneously (intermediate aberrant methylation). Thus, by accounting for both overall mean DNA methylation and epigenetic instability, ‘pure’ hyper‐ and hypomethylated subtypes can be identified. Such subtypes are composed by epigenetically unstable tumors (with high DMI scores) mostly due to hyper‐ or hypomethylation events, as shown for the pan‐GI hypermethylated subtype 5 and pan‐GI hypomethylated subtype 3, respectively. Moreover, DMI analyses contribute to refine the characterization of the intermediate subtypes—lower GI and pan‐GI intermediate subtypes 2 and 6—displaying high DMI scores due to intermediate degrees of both hyper‐ and hypomethylation.

### 3.5 Subtypes associate with clinical features and known GI molecular phenotypes

We mapped the six subtypes against clinical annotations provided by TCGA and GEO for individual samples. Significant associations were found between subtypes and patient gender (*P‐*value = 0.0005) or tumor stage (*P‐*value = 0.01) (Table S2). Some subtypes also showed statistically significant differences regarding age (Fig. S7), with the lowest median age observed for the lower GI hypomethylated subtype 1 and pan‐GI normal‐like subtype 4.

As vast hypermethylation of CpG islands characterizes CIMP tumors [39], the status for each sample was determined. The results are presented in Figs 1E, 3F and show a significant association between the subtypes and CIMP (*P‐*value = 0.0005). The pan‐GI hypomethylated subtype 3 and pan‐GI normal‐like subtype 4 included a large proportion of CIMP– tumors, whereas pan‐GI hypermethylated subtype 5 and pan‐GI intermediate subtype 6 mostly consisted of CIMP+ and CIMPi samples. Among the lower GI tract‐specific subtypes, lower GI hypomethylated subtype 1, and lower GI intermediate subtype 2, there was a predominance of CIMP– and CIMPi samples, respectively. Of these two, only the lower GI intermediate subtype 2 included CIMP+ tumors, which comprised 24% of all colon and rectal tumors located in this subtype. Higher mean methylation, higher frequency of hypermethylated probes, and lower frequency of hypomethylated probes associated with CIMP+ (*P*‐values <0.01, by one‐way ANOVA test). Moreover, an association was found between higher DMI and CIMP+ (*P*‐values = 0.04, by one‐way ANOVA test) (Fig. 3E).

Associations between CIMP+ tumors and *MLH1* silencing via promoter methylation have been well described in CRC [47]. The distribution of *MLH1* promoter methylation status across the different GI subtypes is shown in Fig. 1E and Fig. S8. A statistically significant association between CIMP+ and *MLH1* promoter hypermethylation status was found (*P*‐value = 0.00050, by Fisher's exact test). All normal samples were found to be CIMP– and none showed *MLH1* hypermethylation (data not shown).

### 3.6 Consensus DNA methylation‐based subtypes have independent prognostic outcome

**Fig. 4:**
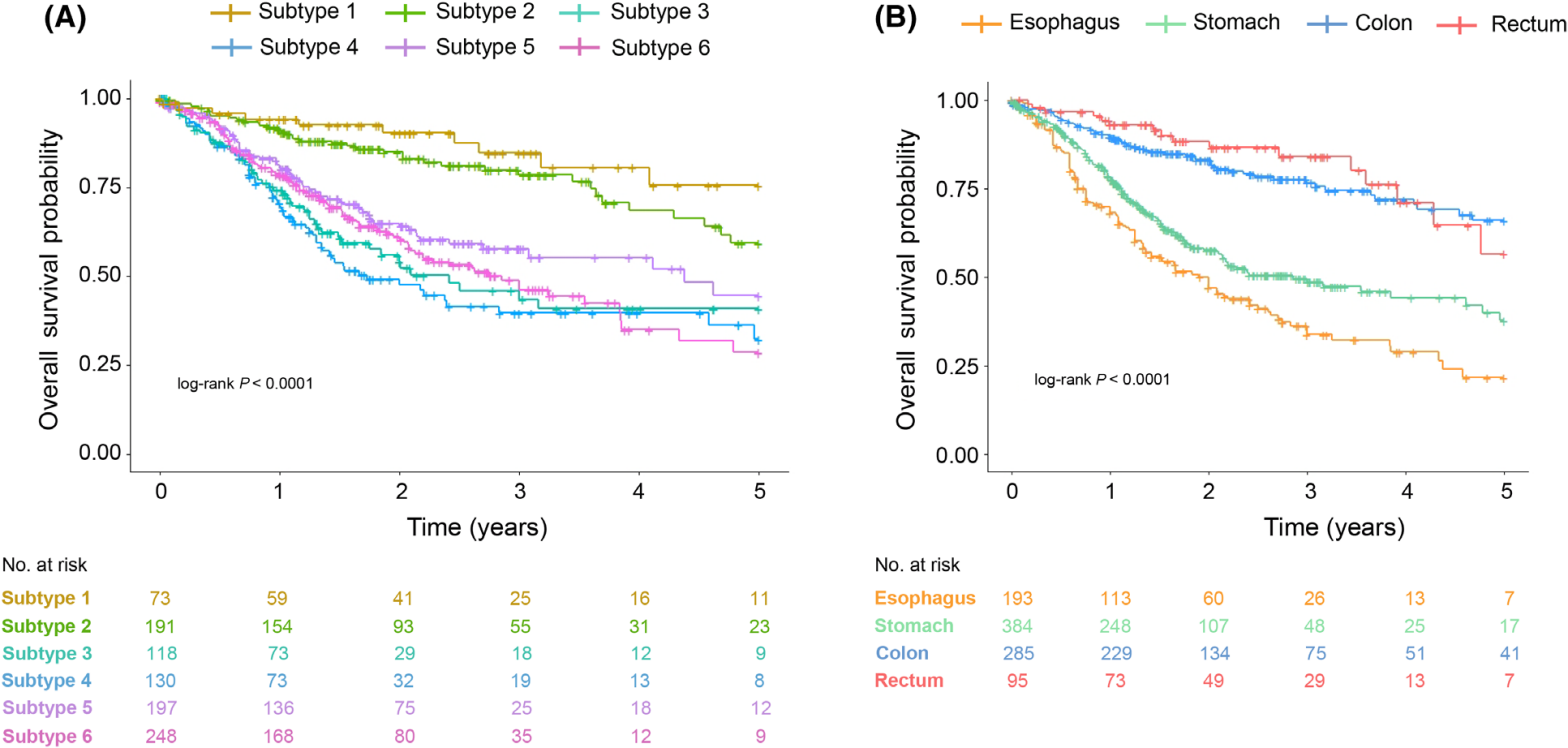
Overall survival modeled by the Kaplan–Meier method and compared using the log‐rank test. (A) Effect of DNA methylation‐based subtypes. (B) Effect of tumor type.

The DNA methylation‐based GI subtypes were significantly associated with overall survival (*P* < 0.001, by log‐rank test; Fig. 4A). The significance of this association was confirmed upon splitting of the GI dataset into a discovery and a validation set (*p* = 0.00075 and *P* < 0.0001, respectively, by log‐rank test). Compared to patients in the lower GI hypomethylated subtype 1, patients in all the other subtypes showed a significant worse prognosis, except for the lower GI intermediate subtype 2 (Table 1). Interestingly, patients belonging to the pan‐GI normal‐like subtype 4 showed the worst prognosis (Fig. 4A; Table 1). Localization was also significantly associated with overall survival (P < 0.001, by log‐rank test; Fig. 4B), and a significantly better prognosis was observed for patients with colon and rectal tumors (Table 1). The same trend was observed upon stratification of the patients into upper and lower GI tract tumors instead or organ of origin. CIMP status and DMI scores did not seem to have any effect on patient overall survival, contrarily to male gender, higher tumor stage, older age (≥75 years), and *MLH1* negative promoter hypermethylation, which were significantly associated with worse overall survival.

**Table 1:** Univariate and multivariate Cox proportional hazard analyses after variable selection procedure by Akaike information criterion, with overall survival as end point. Data from 957 out of 978 patients were available for univariate analysis and a total of 740 patients had information on all best‐fitting predictor variables and therefore included in the multivariate analyses. Due to known associations with patient prognosis, CIMP status was also included in the final model even though not selected by Akaike information criterion. A *P*‐value < 0.05 was considered significant (in bold). CI, confidence interval; CIMP, CpG island methylator phenotype; DMI, DNA methylation instability; GI, gastrointestinal; HR, hazard ratio.

	Patients, *n*	Events, *n*	Univariate HR (95% CI)	*P*‐value	Events, *n*	Multivariate HR (95% CI)	*P*‐value
Subtype							
1 – Lower GI hypomethylated	73	10	1.00 (ref)		9	1.00 (ref)	
2 – Lower GI intermediate	191	40	1.6 (0.8–3.2)	0.178	37	1.54 (0.71–3.31)	0.274
3 – Pan‐GI hypomethylated	118	49	4.5 (2.3–8.9)	**<0.001**	40	2.29 (1.01–5.15)	**0.046**
4 – Pan‐GI normal‐like	130	62	5.2 (2.7–10.2)	**<0.001**	46	3.18 (1.40–7.25)	**0.006**
5 – Pan‐GI hypermethylated	197	69	3.3 (1.7–6.5)	**<0.001**	46	1.64 (0.68–3.95)	0.273
6 – Pan‐GI intermediate	248	103	4.2 (2.2–8.0)	**<0.001**	63	1.93 (0.84–4.41)	0.119
Localization							
Esophagus	193	110	1.00 (ref)		30	1.00 (ref)	
Stomach	384	148	0.69 (0.54–0.88)	**0.003**	142	0.73 (0.48–1.12)	0.151
Colon	285	59	0.27 (0.20–0.37)	**<0.001**	56	0.38 (0.22–0.65)	**<0.001**
Rectum	95	16	0.22 (0.13–0.38)	**<0.001**	13	0.26 (0.12–0.57)	**<0.001**
Gender							
Male	626	245	1.00 (ref)		160	Not included	
Female	331	88	0.65 (0.51–0.83)	**<0.001**	81	Not included	
Age							
<60	303	91	1.00 (ref)			1.00 (ref)	
60–74	430	156	1.30 (0.98–1.60)	0.069	56 117	1.86 (1.34–2.58)	**<0.001**
≥75	215	83	1.40 (1.02–1.80)	**0.037**	68	2.28 (1.57–3.33)	**<0.001**
Stage							
I	114	15	1.00 (ref)		15	1.00 (ref)	
II	283	63	1.80 (1.00–3.10)	**0.046**	63	1.80 (1.02–3.17)	0.043
III	310	120	3.50 (2.00–6.00)	**<0.001**	119	3.34 (1.94–5.74)	**<0.001**
IV	90	44	5.20 (2.90–9.30)	**<0.001**	44	6.82 (3.75–12.41)	**<0.001**
CIMP							
CIMP–	275	96	1.00 (ref)		76	1.00 (ref)	
CIMPi	369	126	0.95 (0.73–1.20)	0.713	90	0.92 (0.65–1.31)	0.655
CIMP+	313	111	1.05 (0.80–1.40)	0.741	75	1.06 (0.68–1.63)	0.800
*MLH1*							
Unmethylated	847	307	1.00 (ref)		215	1.00 (ref)	
Methylated	110	26	0.61 (0.41–0.91)	**0.016**	26	0.71 (0.45–1.12)	0.142
DMI score^a^							
Low	315	118	1.00 (ref)		86	Not included	
High	642	215	0.81 (0.65–1.0)	0.065	155	Not included	

a Subtypes 1 and 4 were considered as having ‘low’ DMI scores, whereas the other subtypes were considered as having ‘high’ DMI scores.

The pan‐GI hypomethylated subtype 3 and the pan‐GI normal‐like subtype 4 remained significantly associated to a worse overall survival in the multivariate Cox's regression analyses, in addition to low GI tract tumor localization, older age (≥60) and higher tumor stage (III and IV) (Table 1).

## 4 Discussion

**Fig. 5:**
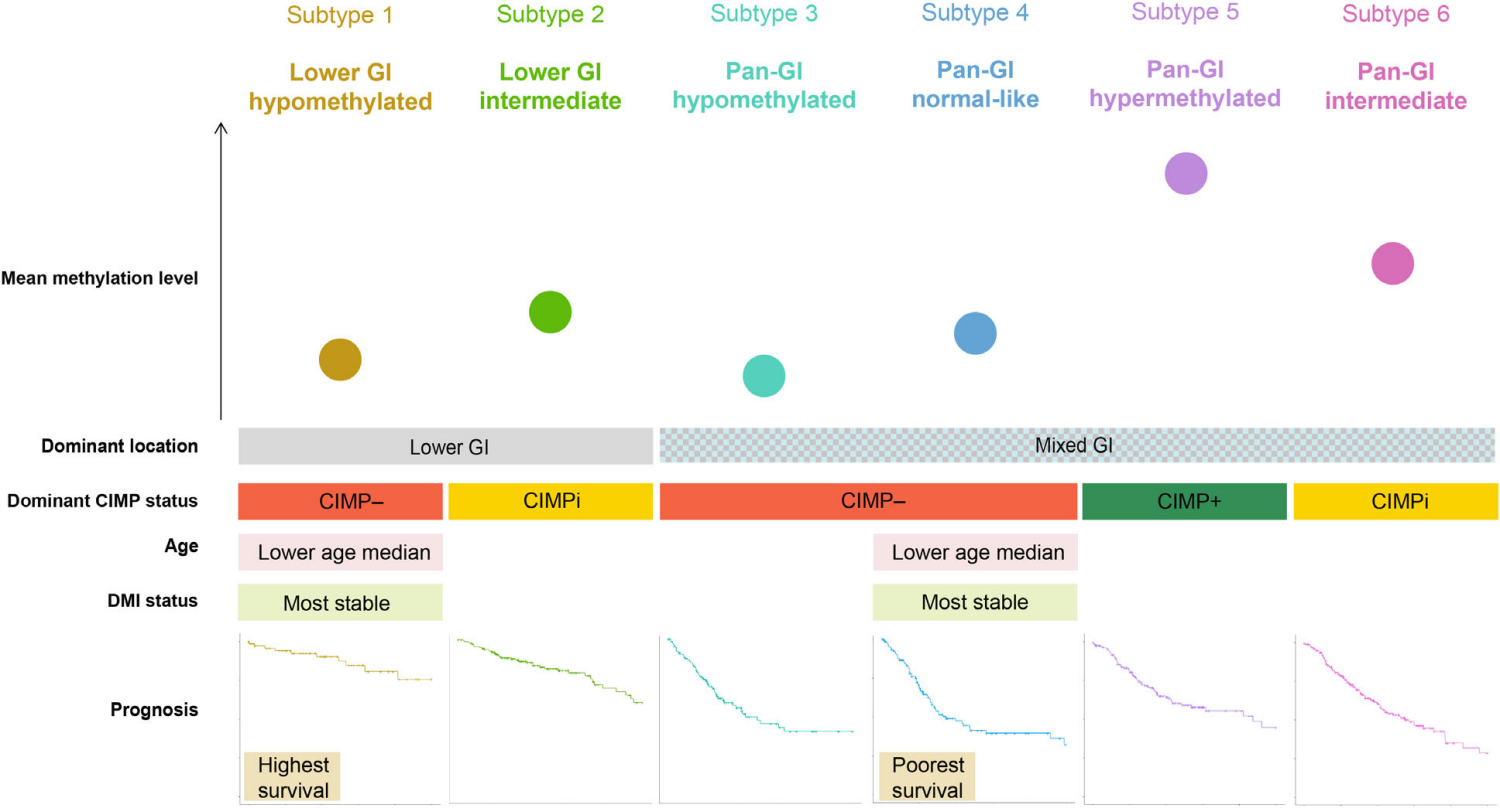
Overview of molecular and clinical features characterizing the six gastrointestinal (GI) subtypes identified. Differences in relative mean methylation levels, dominant location, CpG island methylator phenotype (CIMP), and DNA methylation instability (DMI) status of the tumors, as well as patient age and overall survival curves, are shown. CIMPi, CIMP intermediate.

In this study, we conducted a comprehensive analysis of the DNA methylation patterns in GI adenocarcinomas, presenting evidence for an improved epigenetic characterization and prognostic assessment of these tumors. We proposed a classification into six distinct subtypes defined either by pan‐GI or a lower GI‐specific phenotype. These subtypes displayed unique methylation profiles associated with specific molecular and clinical features, as outlined in Fig. 5.

In contrast to transcriptome‐based analyses, standardized approaches and consistent workflows to identify DNA methylation‐based subtypes are not fully established in the literature. This challenges the description of epigenetic heterogeneity across GI adenocarcinomas and the assessment of their clinical relevance. Our study relied on a robust bioinformatics and statistical framework (Fig. S1), which included a thorough sample and probe selection, as well as an adequate normalization procedure and a rigorous sample clustering method. We expanded the TCGA cohort by adding a GEO dataset [12], in order to ensure a sufficient number of normal gastric samples to perform robust differential analyses, which has remained a challenge in other studies using gastric samples from TCGA only [39]. Feature selection for clustering was based on differential variability between tumor and normal samples as an alternative to traditional feature selection methods to capture the variation in methylation across samples [45]. In contrast, analysis of differential methylation, as used in other studies [8, 16, 39], may lead to preferential selection of methylated probes *vs*. unmethylated probes. DNA methylation variability has been considered in previous studies, for example, defining CIMP. However, such approaches are often based on the top most variable probes across tumors [12, 13, 14, 15] and therefore lack an appropriate hypothesis testing framework.

Methylation level was the main driver of sample clustering, in agreement with previous studies showing that DNA methylation‐based stratification tend to group GI adenocarcinomas by CIMP status rather than by organ of origin [16]. Our stratification further enabled to distinguish subtypes which otherwise would be classified as having the same CIMP status enrichment. This was the case of the lower GI hypomethylated subtype 1, the pan‐GI hypomethylated subtype 3 and the pan‐GI normal‐like subtype 4, all enriched in CIMP– tumors. Our approach therefore suggests that there is room for refining CIMP stratification at a pan‐GI level. CIMP, as defined by the five‐marker panel proposed by Wiesenberger, is well described in CRC [48]. Multiple studies—including the one we used for CIMP classification—have also attempted to stratify tumor samples according to CIMP status from array data, both within individual cancer types [11, 12, 13, 14, 15] and at a pan‐cancer level [16, 39]. However, to date, no consistently methylated targets have been identified to represent a generalizable CIMP phenotype across tumor types. In the present study, 24% of the colon and rectal tumors in the lower GI hypermethylated subtype 2 were CIMP+. This is in line with the frequencies of CIMP‐high CRCs found by genome‐scale analyses [11, 15], which are higher than the 15% detected by the Wiesenberger panel. Additional probes present on the arrays possibly contribute to a more accurate identification of CIMP tumors, as previously suggested [15].

In agreement with the known relation between recurrent hypermethylation and tumor subgroups characterized by CIMP [39], we found an association between high mean methylation, high hypermethylation event frequency, or low hypomethylation event frequency and CIMP+. In addition, an association was found between high DMI and CIMP+, suggesting that measuring the DNA methylation level and the degree of epigenetic instability across all CpG sites add another level of complexity which is not available from previously defined methylation subtypes and which goes beyond CIMP stratification.

Importantly, we found significant differences in the prognostic status of patients across the six GI molecular subtypes, with the worst prognosis seen for patients belonging to the pan‐GI normal‐like subtype 4, followed by the patients in the pan‐GI hypomethylated subtype 3. Both subtypes kept a significant worse survival when correcting for age, stage, tumor localization, and CIMP status of the tumor samples. The poor prognosis of patients within pan‐GI normal‐like subtype 4 is in agreement with previous findings [13] showing that esophageal adenocarcinoma patients belonging to a ‘normal‐like’ subtype had lower survival compared with patients in other subtypes. As suggested by Jammula et al. [13], the tumor microenvironment composition of this group of tumors might be the cause for the negative effect on patient overall survival.

Organ of origin also showed a strong association with overall survival. Significantly better prognosis was observed for patients with colon and rectal tumors, consistent with the better prognosis seen for patients within the lower GI tumor‐enriched subtypes. These observations mirror the fact that, in spite of their unifying molecular characteristics, GI adenocarcinomas present highly heterogeneous clinical courses. However, 31% of the colon and rectal tumors included in out GI dataset were placed in the pan‐GI subtypes, with poorer outcome than the lower GI tumor‐enriched subtypes, adding prognostic value to our DNA methylation‐based stratification when compared to localization alone.

We found no effect of CIMP on overall survival. The lack of association is kept when patients are stratified by tumor localization, in line with some studies on CRC [49], but in contrast to others reporting that CIMP status is associated with worse prognosis in esophageal and CRC [12, 50] or with improved survival in some cohorts of gastric cancer patients with CIMP‐high tumors [51]. These discrepancies may be explained by different selection of markers to define CIMP.

When studying epigenetic heterogeneity, it is important to control for factors known to influence the methylome, such as patient age. In gastric and colon cancers, DNA methylation alterations—either hypermethylation of CpG islands or, to an even greater extent, global hypomethylation—are positively correlated with age [52]. In line with this observation, we found that the two subtypes with infrequent aberrant methylation and therefore lower DMI (lower GI hypomethylated subtype 1 and pan‐GI normal‐like subtype 4) also comprised tumors from younger patients (Fig. S7). We cannot discard that the high DNA methylation levels detected in the pan‐GI hypermethylated and intermediate subtypes 5 and 6 are at least partially attributable to age.

A previous array‐based methylation profiling [8] classified GI adenocarcinomas into seven molecular subtypes: EBV‐CIMP, CIMP‐high, gastroesophageal adenocarcinoma‐CIMP‐low, CRC‐CIMP‐low, and three non‐CIMP subtypes. Although Liu et al. have excluded tissue‐specific DNA methylation, a stronger influence of organ of origin methylation patterns was detected when compared to our study. However, differences in the grounding statistical framework make our sample stratification not directly comparable. Namely, the authors have based their analyses on β‐values and used binary distance between tumor samples in the unsupervised hierarchical clustering. Furthermore, feature selection was exclusively conducted on probes mapped to gene promoters and based on differences in the methylation levels between tumor and normal samples.

Genome‐wide DNA methylation‐based subtyping such as the one described in our study may represent an important initial step toward the identification of clinical relevant biomarkers. Further work is needed in order to find and validate individual candidate subtype‐specific features that can be implemented in clinical practice. Given the association of our subtypes with overall survival, such features could be used for patient prognostication. Moreover, integration with transcriptomic data would allow detecting biologically relevant methylated sites in each subtype, thus providing insight into more targeted treatment recommendations.

## 5 Conclusions

Our analytical framework provided an alternative way to look at the methylome of GI adenocarcinomas, contributing to a more complete picture of epigenetic heterogeneity beyond existing molecular classifications. It identified six distinct methylation‐based subtypes with prognostic value, independent of the organ of origin, and without limiting the analysis to hypermethylation at CpG islands. Our approach may represent a relevant setting for systematic investigation of aberrant DNA methylation patterns and it can be further exploited as a potential reference for future biomarker selection for patient stratification, offering guidance for prognostic evaluations, and therapeutic strategies planning.

## Conflict of interest

The authors declare no conflict of interest.

## Author contributions

RP: conceptualization, data curation, formal analysis, investigation, methodology, software, visualization, data interpretation, and writing (original draft); HMV: data interpretation and writing (review and editing); GEL: data interpretation, funding acquisition, resources, and writing (review and editing); MJ: conceptualization, data interpretation, funding acquisition, methodology, project administration, resources, software, supervision, validation and writing (review and editing). All authors contributed to the article and approved the final version.

### Peer review

The peer review history for this article is available at https://www.webofscience.com/api/gateway/wos/peer‐review/10.1002/1878‐0261.13772.

## Supporting information


**Fig. S1.** Overview of data preparation and analyses performed in this study.
**Fig. S2.** Effect of functional normalization in the methylation data.
**Fig. S3.** Differentially variable probes found for each tumor type when compared to respective normal samples.
**Fig. S4.** Consensus plots obtained from hierarchical clustering based on methylation data of 978 GI adenocarcinomas using ConsensusClusterPlus package for selection of the optimal number of subtypes.
**Fig. S5.** Epigenetic heterogeneity across the six subtypes.
**Fig. S6.** Methylation aberrancy across the subtypes.
**Fig. S7.** Distribution of age across samples for each subtype.
**Fig. S8.** Distribution of *MLH1* promoter hypermethylation status across subtypes.
**Table S1.** Overview of the number of samples analyzed in this study and of significant differentially variable probes found for each tumor type.
**Table S2.** Associations between the six subtypes and clinical features.

## Data Availability

The data used in this study were downloaded from GEO (https://www.ncbi.nlm.nih.gov/geo/) under accession number GSE72872 and from Genomic Data Commons portal (https://portal.gdc.cancer.gov) under restricted access (dbGaP accession number: phs000178.v11.p8).
