# Supplementary material for: Unraveling epigenetic heterogeneity across gastrointestinal adenocarcinomas through a standardized analytical framework
Source: Mol Oncol. 2024 Dec 18;19(4):1117–31. doi: 10.1002/1878-0261.13772 (PMC11977639; doi:10.1002/1878-0261.13772)
Supplement: Supplementary file 1 — Fig. S1. Overview of data preparation and analyses performed in this study. Fig. S2. Effect of functional normalization in the methylation data. Fig. S3. Differentially variable probes found for each tumor type when compared to respective normal samples. Fig. S4. Consensus plots obtained from hierarchical clustering based on methylation data of 978 GI adenocarcinomas using ConsensusClusterPlus package for selection of the optimal number of subtypes. Fig. S5. Epigenetic heterogeneity across the six subtypes. Fig. S6. Methylation aberrancy across the subtypes. Fig. S7. Distribution of age across samples for each subtype. Fig. S8. Distribution of MLH1 promoter hypermethylation status across subtypes. Table S1. Overview of the number of samples analyzed in this study and of significant differentially variable probes found for each tumor type. Table S2. Associations between the six subtypes and clinical features. [file MOL2-19-1117-s001.docx]

**Supporting Information for**

**Unraveling epigenetic heterogeneity across gastrointestinal adenocarcinomas through a standardized analytical framework**

Rita Pinto, Hege Marie Vedeld, Guro Elisabeth Lind, Marine Jeanmougin

**Contents:**

Figures S1-S8

Tables S1-S2

**Fig. S1.** Overview of data preparation and analyses performed in this study.


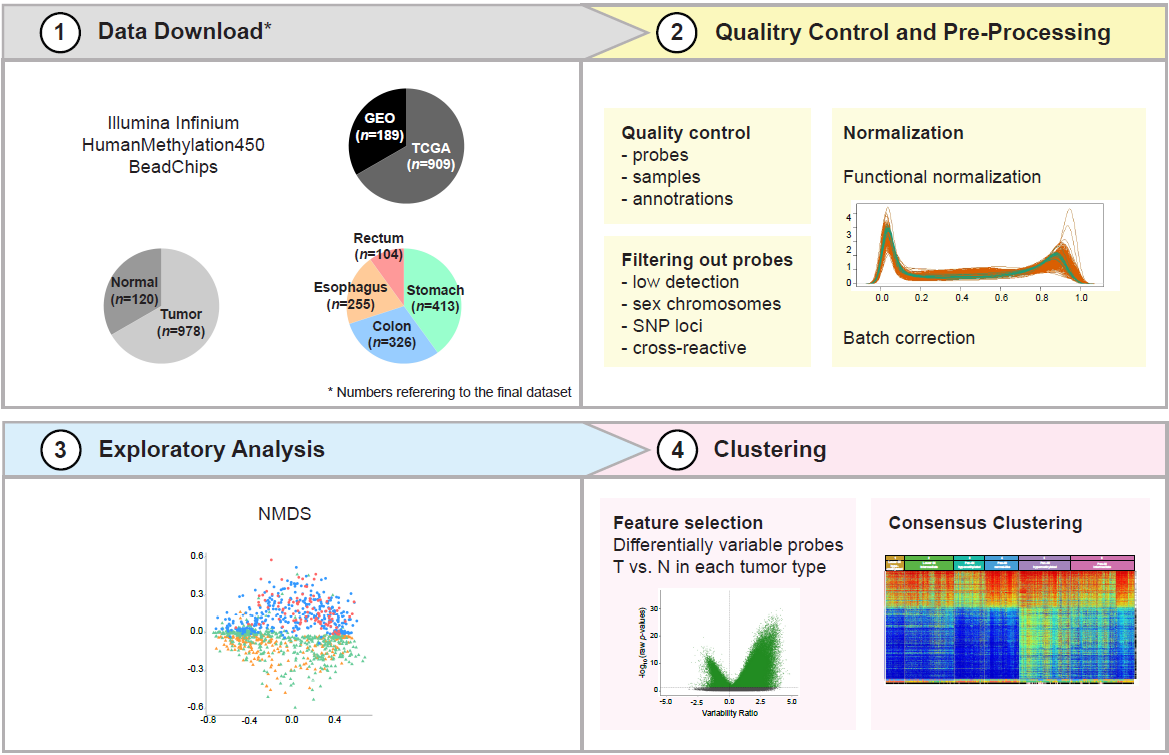


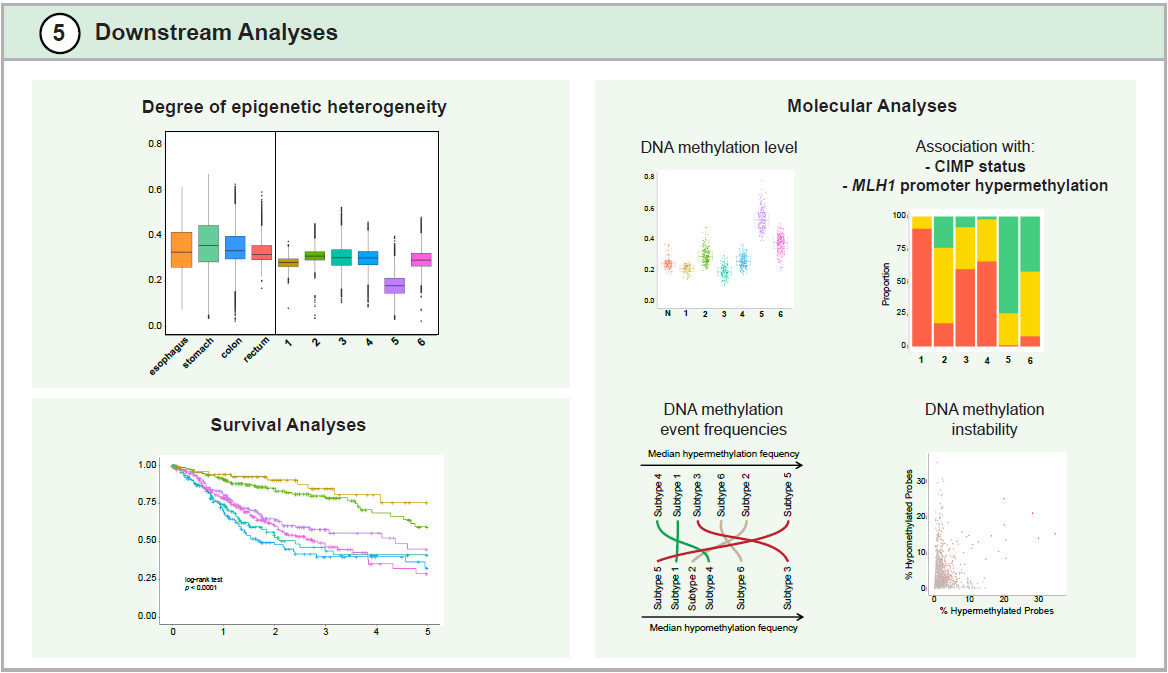


**Fig. S2.** Effect of functional normalization in the methylation data. Normal samples are represented in green and tumor samples in orange.


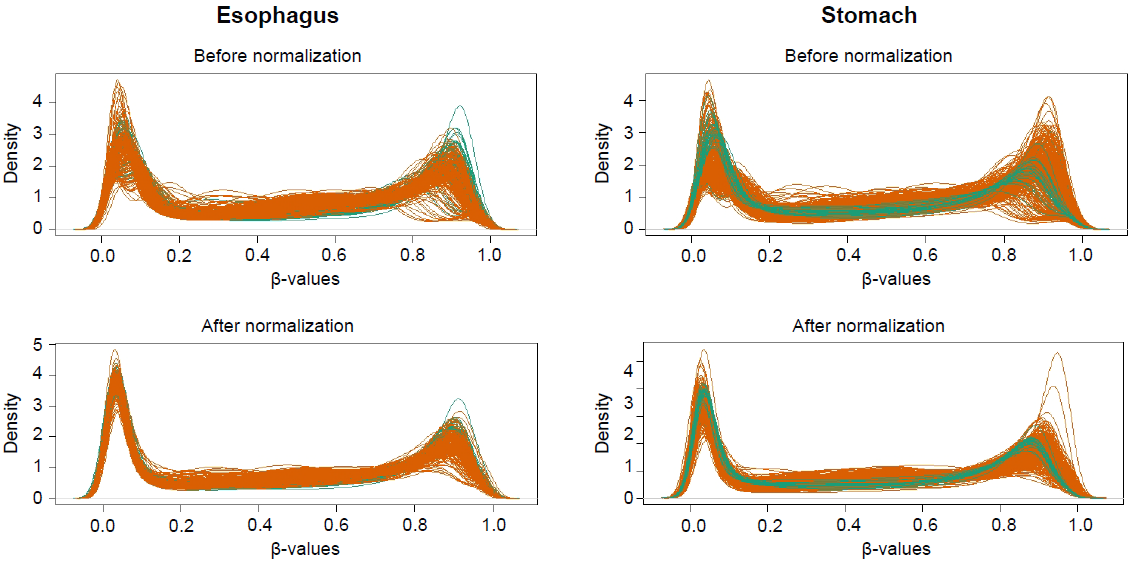


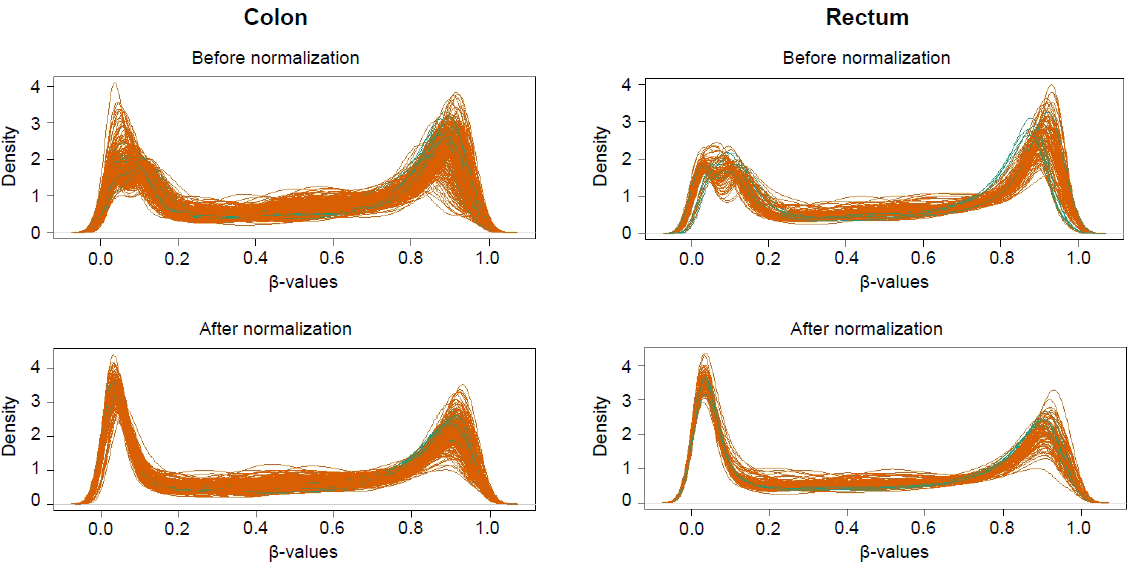


**Fig. S3.** Differentially variable probes (DVPs) found for each tumor type when compared to respective normal samples. (A) Venn diagram shows the distribution of significant tumor-specific or common DVPs across tumor types. (B) Volcano plots show the original *p*-values as a function of the variability ration for each probe. All probes for which raw *p*-value > 0.05 are depicted in green.


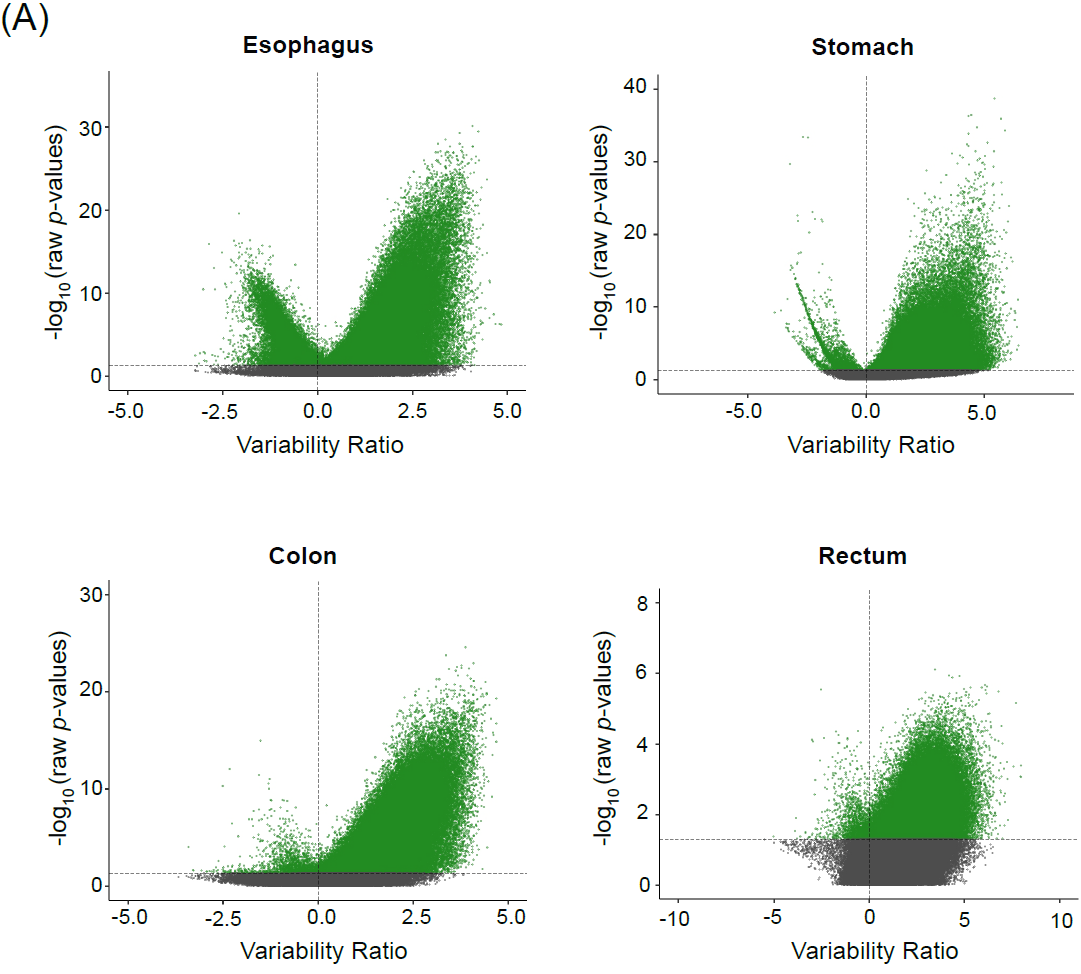


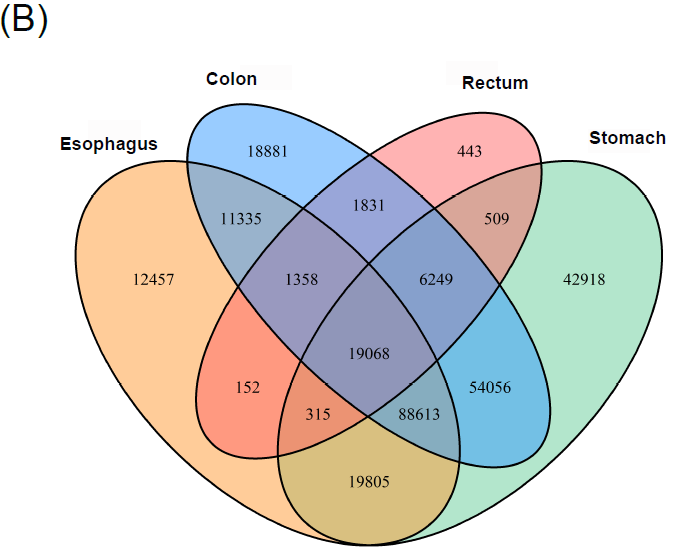


**Fig. S4.** Consensus plots obtained from hierarchical clustering based on methylation data of 978 GI adenocarcinomas using ConsensusClusterPlus package for selection of the optimal number of subtypes. (A) Cumulative distribution function (CDF) curves. (B) Relative change in the area under CDF curves (k = 2 to k = 10, where k denotes the number of clusters/subtypes). (C) Dendrogram and consensus matrix. The intensity of the blue color is proportional to the frequency at which samples have been clustered together.


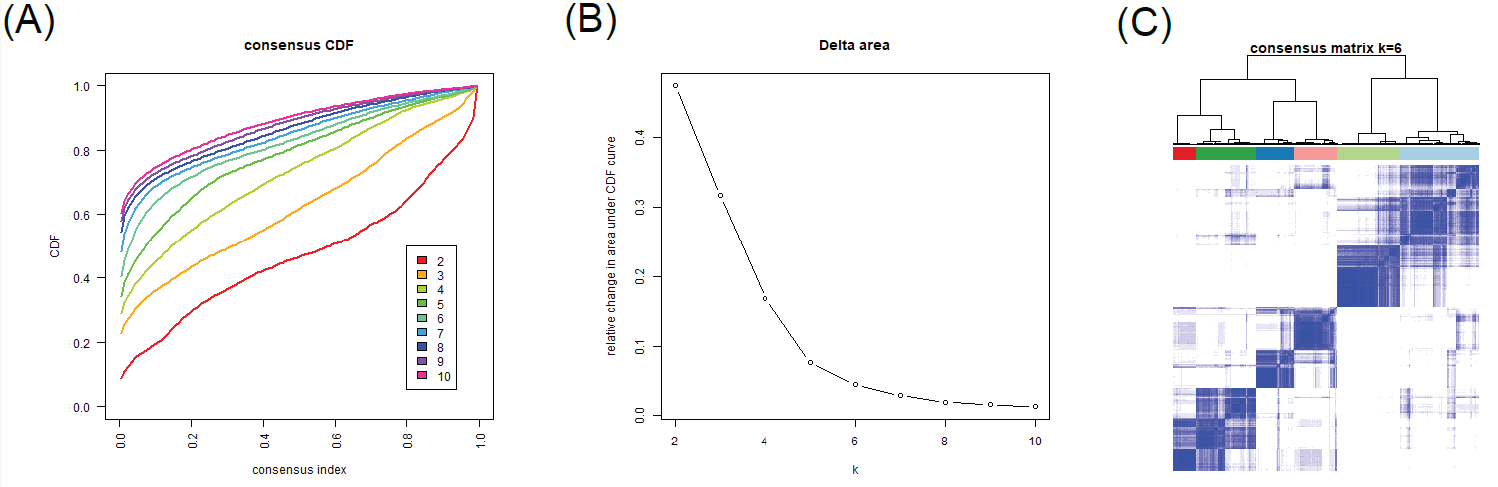


**Fig. S5.** Epigenetic heterogeneity across the six subtypes. (A) Non-metric multidimensional scaling (NMDS) plots based on the union of the 1% most significant DVPs found for each tumor type, as in Fig. 1A,B. Each dot represents one sample colored by subtype. (B) Correlation between the first axis of NMDS and the mean methylation for each tumor sample, as in Fig. 1C.


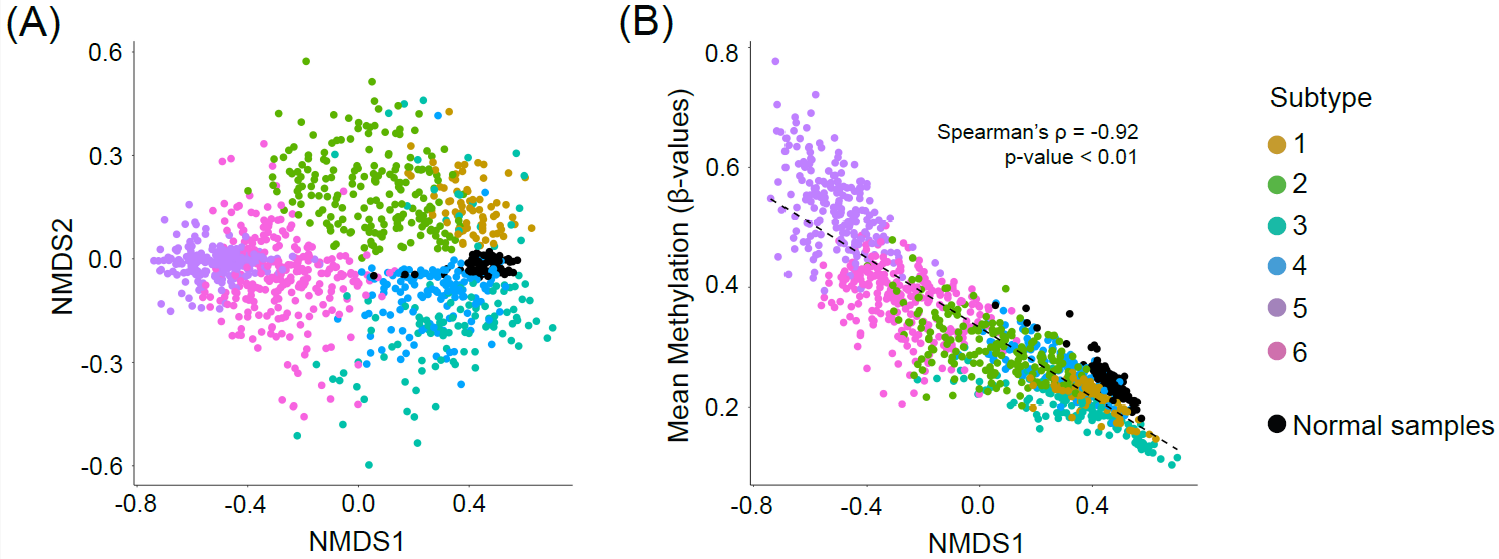


**Fig. S6.** Methylation aberrancy across the subtypes. Both hyper- (A) and hypomethylation (B) frequencies are depicted. Frequencies were estimated based on the RESET algorithm established by Saghafinia *et al*.^1^ Each dot represents one sample and it is colored according to each subtype. The samples within each subtype are sorted by aberrant methylation frequency and the horizontal lines define the frequencies median.


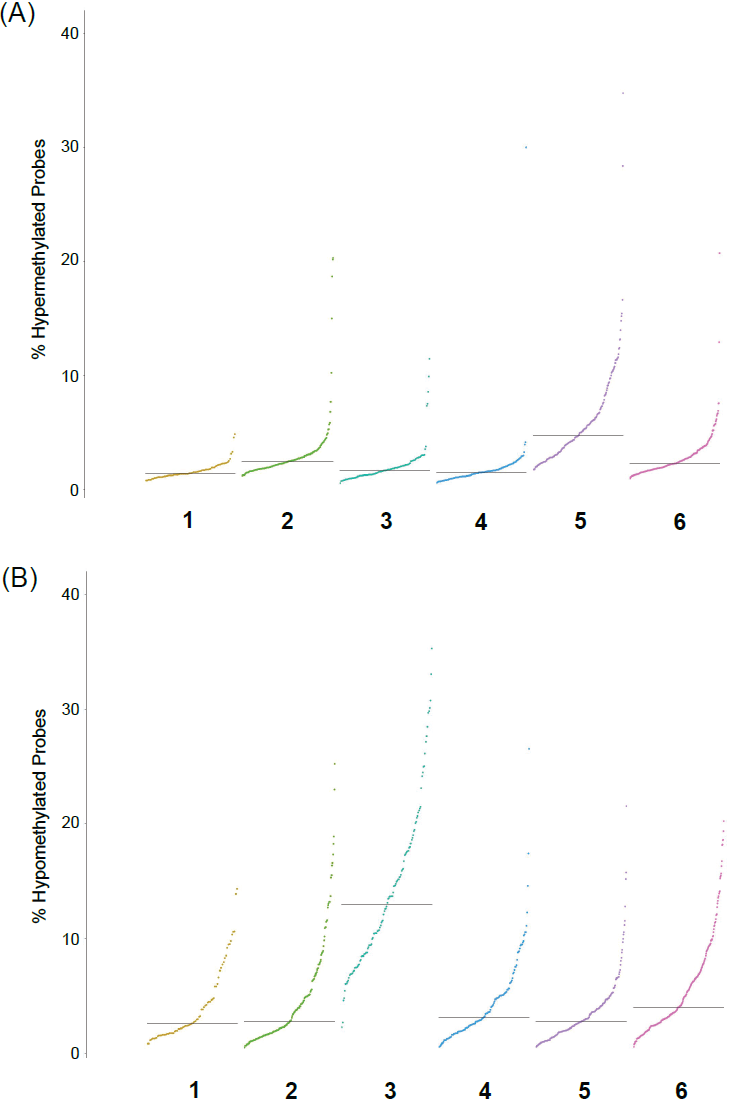


**Fig. S7.** Distribution of age across samples for each subtype. Differences between the subtypes were evaluated by Tukey Honest Significant Differences Test. (*adjusted *p*-values < 0.01 were considered statistically significant)


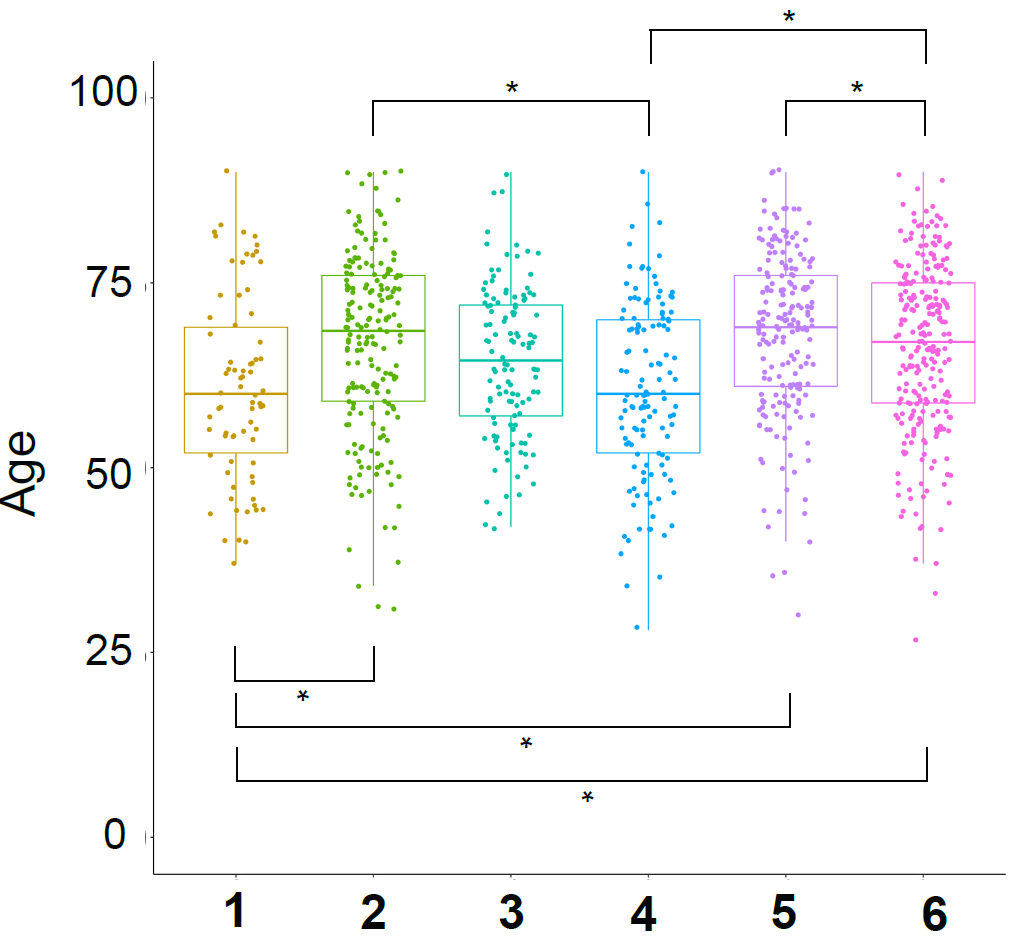


**Fig. S8.** Distribution of *MLH1* promoter hypermethylation status across subtypes.


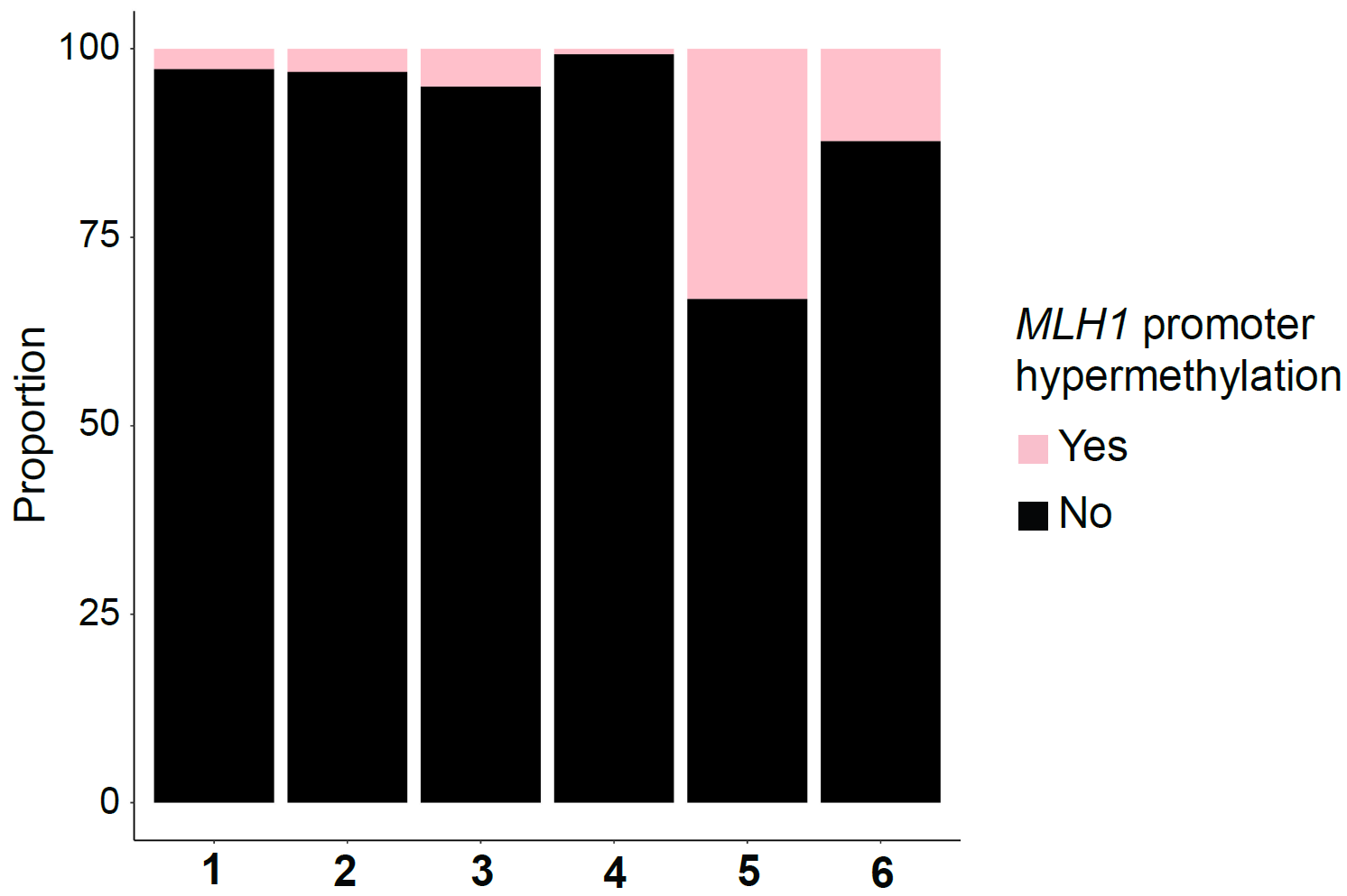


**Table S1.** Overview of the number of samples analyzed in this study and of significant differentially variable probes (DVPs) found for each tumor type. If the methylation variability ratio of a DVP was higher in tumor than in normal samples, we defined it as hypervariable probe, otherwise it was considered as a hypovariable probe. T: tumor; N: normal; DVP: differentially variable probe.

|  | **TCGA dataset** | **GEO dataset** | **Total** | **Significant DVPs T *vs* N (FDR < 0.05)** |
| --- | --- | --- | --- | --- |
| **Esophagus** | **T:** 76  **N:** 11 | **T:** 125  **N:** 43 | **T:** 201  **N:** 54 | **Total:** 152 694  **Hypovariable:** 21 573 (14%)  **Hypervariable:** 131 121 (86%) |
| **Stomach** | **T:** 392  **N:** 0 | **T:** 0  **N:** 21 | **T:** 392  **N:** 21 | **Total:** 232 124  **Hypovariable:** 2 958 (1%)  **Hypervariable:** 229 166 (99%) |
| **Colon** | **T:** 288  **N:** 38 | **T:** 0  **N:** 0 | **T:** 288  **N:** 38 | **Total:** 201 982  **Hypovariable:** 1 823 (1%)  **Hypervariable:** 200 159 (99%) |
| **Rectum** | **T:** 97  **N:** 7 | **T:** 0  **N:** 0 | **T:** 97  **N:** 7 | **Total:** 30 516  **Hypovariable:** 759 (2%)  **Hypervariable:** 29 757 (98%) |
| **Total** | **T:** 853  **N:** 56 | **T:** 125  **N:** 64 | **T:** 978  **N:** 120 |  |

**Table S2.** Associations between the six subtypes and clinical features.

|  | **Total, *n*** | **Subtype 1,**  ***n* (%)** | **Subtype 2,**  ***n* (%)** | **Subtype 3,**  ***n* (%)** | **Subtype 4,**  ***n* (%)** | **Subtype 5,**  ***n* (%)** | **Subtype 6,**  ***n* (%)** | ***p*-value** |
| --- | --- | --- | --- | --- | --- | --- | --- | --- |
| **Number of patients** | 978 | 74 (7%) | 194 (20%) | 120 (12%) | 135 (14%) | 202 (21%) | 253 (26%) |  |
| **Gender**  Male  Female  NA | 636  333  9 | 35 (5%)  38 (11%)  1 (11%) | 106 (17%)  86 (26%)  2 (22%) | 84 (13%)  35 (11%)  1 (11%) | 86 (13%)  47 (14%)  2 (22%) | 137 (22%)  64 (19%)  1 (11%) | 188 (30%)  63 (19%)  2 (22%) | **0.0005** |
| **Stage**  I  II  III  IV  NA | 118  284  313  90  173 | 8 (7%)  22 (8%)  26 (8%)  15 (17%)  3 (2%) | 28 (24%)  73 (26%)  57 (18%)  24 (27%)  12 (7%) | 15 (13%)  35 (12%)  40 (13%)  14 (15 %)  16 (9%) | 11 (9%)  27 (10%)  46 (15%)  13 (14%)  38 (22%) | 33 (28%)  58 (20%)  69 (22%)  7 (8%)  35 (20%) | 23 (19%)  69 (24%)  75 (24%)  17 (19%)  69 (40%) | **0.01** |

**Supplementary References**

1. Saghafinia S, Mina M, Riggi N, Hanahan D, Ciriello G. Pan-Cancer Landscape of Aberrant DNA Methylation across Human Tumors. *Cell Reports*. 2018;25(4):1066-1080.e8. doi:10.1016/j.celrep.2018.09.082
